# In Vitro Drug Release, Permeability, and Structural Test of Ciprofloxacin-Loaded Nanofibers

**DOI:** 10.3390/pharmaceutics13040556

**Published:** 2021-04-15

**Authors:** Luca Éva Uhljar, Sheng Yuan Kan, Norbert Radacsi, Vasileios Koutsos, Piroska Szabó-Révész, Rita Ambrus

**Affiliations:** 1 Interdisciplinary Excellence Centre, Faculty of Pharmacy, Institute of Pharmaceutical Technology and Regulatory Affairs, University of Szeged, Eötvös Street 6, H-6720 Szeged, Hungary; uhljar.luca.eva@szte.hu (L.É.U.); ReveszPiroska@szte.hu (P.S.-R.); 2 School of Engineering, Institute for Materials and Processes, The University of Edinburgh, King’s Buildings, Edinburgh EH9 3FB, UK; skyshengyuan@hotmail.com (S.Y.K.); n.radacsi@ed.ac.uk (N.R.); vasileios.koutsos@ed.ac.uk (V.K.)

**Keywords:** amorphous solid dispersion, ciprofloxacin, electrospinning, nanofibers, poorly water-soluble drug, povidone

## Abstract

Nanofibers of the poorly water-soluble antibiotic ciprofloxacin (CIP) were fabricated in the form of an amorphous solid dispersion by using poly(vinyl pyrrolidone) as a polymer matrix, by the low-cost electrospinning method. The solubility of the nanofibers as well as their in vitro diffusion were remarkably higher than those of the CIP powder or the physical mixture of the two components. The fiber size and morphology were optimized, and it was found that the addition of the CIP to the electrospinning solution decreased the nanofiber diameter, leading to an increased specific surface area. Structural characterization confirmed the interactions between the drug and the polymer and the amorphous state of CIP inside the nanofibers. Since the solubility of CIP is pH-dependent, the in vitro solubility and dissolution studies were executed at different pH levels. The nanofiber sample with the finest morphology demonstrated a significant increase in solubility both in water and pH 7.4 buffer. Single medium and two-stage biorelevant dissolution studies were performed, and the release mechanism was described by mathematical models. Besides, in vitro diffusion from pH 6.8 to pH 7.4 notably increased when compared with the pure drug and physical mixture. Ciprofloxacin-loaded poly(vinyl pyrrolidone) (PVP) nanofibers can be considered as fast-dissolving formulations with improved physicochemical properties.

## 1. Introduction

Nowadays, one of the main challenges in pharmaceutical technology is dealing with poorly water-soluble drugs. The number of Biopharmaceutics Classification System (BCS) Class II and Class IV drugs is estimated to make up ~40% of currently marketed drugs and ∼90% of compounds currently under development [1,2,3]. One of the BCS Class II/IV drugs is ciprofloxacin (CIP), which is a worldwide-used, broad-spectrum, second-generation fluoroquinolone antibiotic. Fluoroquinolones inhibit DNA replication in bacteria because their mechanism of action is the inhibition of bacterial DNA gyrase and topoisomerase IV. The lack of cross-resistance between fluoroquinolones and other classes of antibiotics makes this antibiotic family important. CIP and its derivatives can effectively treat antibiotic-resistant infections (e.g., nosocomial pneumonia), so they are usually reserved as “drugs of last resort” [4]. Since CIP is active against many Gram-negative and Gram-positive bacteria, it is directed to treat several bacterial infections. CIP is indicated for a broad spectrum of diseases, from bacterial conjunctivitis through bone and joint infections to complicated urinary tract infections. Accordingly, the available dosage forms on the market are ophthalmic and otic solutions, film-coated tablets, oral suspensions, and solutions for infusions. Thus, various routes for drug administration, both local and systemic, are applied. Despite common use, CIP has only 56–77% bioavailability [4]. The solubility of the drug is pH-dependent, as CIP is highly soluble below pH 5 and above pH 10 but almost insoluble around neutral pH level [5]. According to the BCS, CIP belongs to Class IV, with low water solubility (0.067 mg/mL at 25 °C, pH 7.5; [6]) and poor permeability [4].

In most cases, nanocarrier-based drug delivery systems can offer a solution to the low solubility problem of a drug. Nanostructures are innovative formulations with nanometer-scale in at least one dimension and a large surface-area-to-volume ratio. The latter ensures that a large amount of the drug can come into contact with the surrounding medium. In this way, the dissolution rate and even solubility can increase [7,8,9]. Furthermore, numerous drug delivery systems contain the active pharmaceutical ingredient (API) in an amorphous state and therefore cause increased solubility of the drug [10,11]. With the increase of solubility and dissolution rate, the bioavailability of the API increases [12,13,14]. Currently, intensive research is underway to formulate CIP into different nanocarriers to treat several diseases. Some examples are collected in Table 1.

Polymeric nanofibers are considered solid dispersions with a large specific surface area that can stabilize drugs in their amorphous state. Thus, polymer-based nanofibers provide an attractive approach for the development of dosage forms due to the enhanced solubility and dissolution rate [25,26]. Nanofibers have a variety of uses currently under investigation in different fields of science. In pharmaceutical and medical fields, at least four applications have to be mentioned: wound dressings, filtration, drug delivery systems, and tissue engineering scaffolds [27,28,29,30,31]. Electrospinning is an effective and inexpensive method for the fabrication of polymeric nanofibers; hence, it is the most widely used technique for nanofiber production in the industry. During the electrospinning procedure, which is generated by a high voltage power supply, the ejection and travel of the polymer-drug fluid jet are induced by the large potential difference between a needle and a collector [32]. As the jet travels towards the collector, the solvent evaporates, and the jet solidifies into nano-sized fibers. It is possible to control the mean fiber diameter and morphology by changing the polymer-drug solution, the applied voltage, the needle–collector distance, the flow rate, the collector speed, or the environmental temperature and humidity [33,34].

Poly(vinyl pyrrolidone) (PVP) is a water-soluble and biocompatible polymer, which is commonly used as a pharmaceutical excipient and food additive [35]. PVP is also widely used as a carrier polymer that enables electrospinning [23,36,37,38,39,40,41,42,43,44,45].

Ciprofloxacin-loaded PVP nanofibers as potential wound dressings have been recently investigated [23,41,44]. However, to the best of our knowledge, the development of a potential per os formulation has not been published yet.

In this article, the preformulation studies of PVP-based nanofiber mats loaded with CIP for per os administration are investigated. The aim of the present study was to increase the water solubility and diffusion of the API and study the in vitro drug release and its kinetics. Additionally, the study aimed to investigate the effect of the polymer–drug solution and the flow rate on the fiber diameter and morphology. Special attention was paid to structural characterization, drug entrapment efficiency, solubility, in vitro dissolution, and in vitro diffusion of the CIP-loaded nanofibrous samples when producing immediate-release nanofibrous formulation, and therefore achieving effective antibiotic therapy.

## 2. Materials and Methods

### 2.1. Materials

Ciprofloxacin base (CIP; Mw = 331.35; purity >98%) was gifted by Teva Pharmaceutical Works Ltd. (Debrecen, Hungary). Polyvinylpyrrolidone (PVP) (Mw = 1,300,000) was obtained from Alfa Aesar (Lancashire, United Kingdom). Ethanol (99.99% purity) and chloroform (99.8% purity) were purchased from Fisher Scientific (Loughborough, United Kingdom).

Phosphate buffer solutions (PBS, pH 7.4 and 6.8) were prepared in-house as follows. To prepare 1 L of the pH 7.4 PBS, 1.44 g disodium phosphate dihydrate (Na_2_HPO_4_ × 2 H_2_O), 0.12 g potassium dihydrogen phosphate (KH_2_PO_4_), 8.00 g sodium chloride (NaCl), and 0.20 g potassium chloride (KCl) were dissolved in approximately 0.9 L of distilled water. Then the pH was adjusted to 7.4 using aqueous solutions of NaOH, bringing the buffer up to a volume of 1 L. For the preparation of 1 L of the pH 6.8 PBS, 6.8 g KH_2_PO_4_ was dissolved in 900 mL of distilled water before mixing 77.00 mL of aqueous solutions of NaOH (0.2 M). The pH was adjusted to 6.8 using the NaOH solution. All used chemicals were purchased from Sigma-Aldrich (Budapest, Hungary).

All other chemicals were analytical grade, and distilled water was used.

### 2.2. Methods

#### 2.2.1. Preparation of the Solutions for Electrospinning

As the first step of the nanofiber preparation, the electrospinning solutions were prepared. PVP powder was dissolved in ethanol, whereas ciprofloxacin was dissolved in chloroform in a separate vessel. Constant stirring was applied to both at room temperature for 24 h to generate homogenous solutions using a magnetic stirrer at 700 rpm. The nominal concentration of the CIP solutions was 1 mg/mL, while the polymer concentrations varied between 5–20% *w*/*v*. The PVP and the drug solutions were mixed in 1:1, 1:2, 1:3 volume ratio, respectively, to yield the requested concentrations. The full details of the samples prepared are listed in Table 2.

#### 2.2.2. Electrospinning Procedure

**Figure 1:**
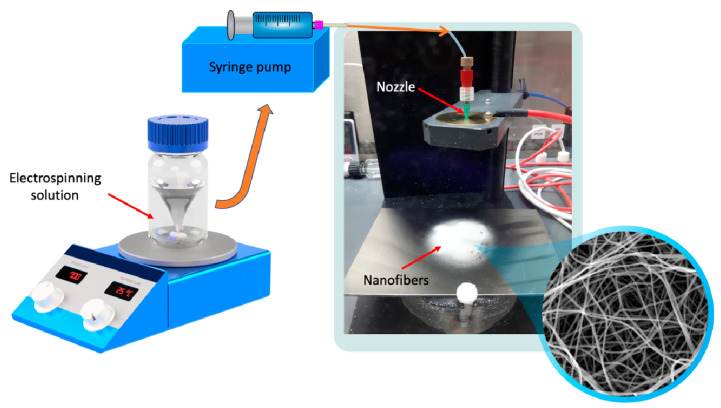
Preparation of ciprofloxacin-loaded nanofibers.

The prepared solutions were filled in 2 mL syringes fitted with stainless steel 20 G needles. The electrospinning process was carried out using a commercially available electrospinning device (IME Medical Electrospinning, Waalre, The Netherlands; Figure 1). The applied potential difference was 24 kV, and the needle-collector distance was maintained at 10 cm. Different flow rates (0.5, 1, 2, 3, and 4 mL/h) were provided by a syringe pump (Table 2). All the experiments were performed at ambient conditions (23 °C temperature and 36–42% relative humidity). Additionally, a control sample containing no CIP was prepared using the same conditions as were used for the other samples (a constant 2 mL/h pump flow rate was used for the pure PVP sample).

#### 2.2.3. Preparation of the Physical Mixture

For the structural characterization and in vitro studies, physical mixtures were used as reference samples, prepared by a shaker mixer (Turbula System Schatz; Willy A. Bachofen AG Maschinenfabrik, Basel, Switzerland). PVP and CIP were homogenized under controlled conditions (50 rpm, 10 min) in the same weight ratio as most nanofibrous samples.

#### 2.2.4. Size and Morphological Measurements

The morphology of the electrospun nanofibers was observed by scanning electron microscopy (SEM; Hitachi S4700, Hitachi Scientific Ltd., Tokyo, Japan) at 10 kV. Specimens of the fiber mats (0.4 × 0.3 cm) were cut and coated with approximately 10 nm thin film of gold–palladium using a sputter coater (Bio-Rad SC 502, VG Microtech, Uckfield, UK) before the SEM imaging was performed. One hundred fibers from each formulation were randomly selected to carry out the fiber diameter measurement (ImageJ 1.44p software; Bethesda, MD, USA).

#### 2.2.5. Structural Characterization

CIP, physical mixture, and CIP-loaded nanofibers were characterized by X-ray powder diffraction (XRPD; D8 Advance, Bruker AXS GmbH, Karlsruhe, Germany). The samples were measured with Cu K λ_I_ radiation (40kV/40mA, λ = 1.5406 Å) in between diffraction angle 3–40° for their structural properties.

Differential scanning calorimeter (DSC; Mettler Toledo DSC821e; Mettler Inc., Schwerzenbach, Switzerland) was applied to evaluate the thermal behavior of the samples using 25–300 °C temperature range at a heating rate of 5 °C min^−1^.

Fourier transform infrared spectroscopy (FTIR; Thermo Nicolet AVATAR 330, Madison, WI, USA) was performed after discs of a KBr and nanofibers had been made by a compression molding technique. The discs were scanned 128 times over the range 4000–400 cm^−1^ and with a resolution of 4 cm^−1^.

#### 2.2.6. Drug Loading and Drug Entrapment Efficiency

The drug loading and the amount of CIP entrapped in the optimized nanofibrous mat was quantified by UV spectrophotometry (ABL&E-Jasco UV/VIS Spectrophotometer V-730, Budapest, Hungary). A known mass of NF6 sample (about 160 mg) was dissolved in 50 mL of 0.1 M hydrochloric acid solution. The amount of CIP in the solution was calculated by UV spectroscopy analysis at a λ_max_ of 277 nm.

The amount of the loaded CIP of the NF6 formulation was calculated by comparing the released mass of CIP (w*_UV_*) with the mass of the dissolved nanofiber mat (w*_UV_*). The drug loading (DL%) was calculated by the following Equation (1).

DL (%) = (w*_UV_*/w*_MAT_*) × 100. (1)

The drug entrapment efficiency of the nanofiber (EE) was calculated by the following Equation (2).

EE (%) = (w*_UV_*/w*_ES_*) × 100, (2)

where w*_UV_* is the calculated mass of CIP released from the nanofibers, and w*_ES_* is the mass of CIP dissolved before the electrospinning procedure.

Each experiment was performed in four parallel measurements, and the average values and standard deviations are reported.

#### 2.2.7. Solubility Tests

Solubility tests were carried out both in distilled water and PBS (pH 7.4) as CIP is a poorly water-soluble drug and has a U-shaped pH-dependent solubility with a minimum around pH 7.4. The first step was to place the pure drug, the physical mixture, and the drug-loaded nanofibrous samples in 3 mL of distilled water (pH 6.3) or PBS (pH 7.4), which were then stirred for 24 h at room temperature with a magnetic bar. The samples were then filtered (0.22 mm, FilterBio PES Syringe Filter; Labex Ltd., Budapest, Hungary), suitably diluted, and measured by UV spectrophotometry (ABL&E-Jasco UV/VIS Spectrophotometer V-730, Budapest, Hungary) at 275 nm and 271 nm in the case of water and PBS, respectively. The solubility tests of each sample were performed in triplicates, and the average values with the standard deviations are reported.

Additionally, the solubility of the CIP was measured with the same methodology in Fasted State Simulating Gastric Fluid (FaSSGF; Biorelevant.com accessed on 6 April 2021, London, UK) and Fasted State Simulating Intestinal Fluid (FaSSIF; Biorelevant.com accessed on 6 April 2021, London, UK) to get data for the in vitro two-stage drug release study. For the UV spectroscopy, 277 nm and 272 nm in the case of the FaSSGF and FaSSIF media were set as the wavelength of light, respectively. The results of the measurements are reported in Section 3.5.

#### 2.2.8. In Vitro Drug Release Studies

Two types of in vitro drug release studies were executed to get a complete picture of the CIP release. Firstly, single medium dissolution (pH 7.4 PBS) with a modified paddle method (Hanson Research SR8-Plus Dissolution Test Station, Chatsworth, CA, USA) was used to measure the drug release from NF6 nanofibers (containing 25 mg CIP) compared with its corresponding physical mixture, and also with 25 mg raw CIP powder. The release studies were carried out in 100 mL of the PBS medium at 37 °C. The paddle was rotated at 100 rpm. Then, 5 mL samples were taken manually from the buffer solution after 5, 10, 15, 30, 60, and 90 min. After sampling, the volume was replaced with fresh PBS. The amount of drug present in the aliquots was determined with UV-Vis spectrophotometry (ABL&E-Jasco UV/VIS Spectrophotometer V-730, Budapest, Hungary) at a λ_max_ of 275 nm. The cumulative CIP release (~25 mg = 100%) was calculated using the calibration curve of CIP in pH 7.4 PBS. Each experiment was performed in triplicate, and the average values and standard deviations are reported.

Besides, to mimic the in vivo conditions better, a two-stage release test as a biorelevant gastrointestinal transfer protocol was also studied by the Hanson dissolution tester mentioned above. A portion of 25 mg CIP powder was compared with a 100-fold larger mass of NF6 electrospun sample as the nanofibers were containing 1 w/w % CIP. The samples were first added to 25 mL of FaSSGF, and 2 mL aliquots were taken at the time points: 1, 3, 5, 10, 15, and 30 min. Right after the last sampling, 25 mL of FaSSIF Concentrate, as a “bolus”, was quickly added to the gastric medium. The FaSSIF Concentrate’s surfactant concentration and buffer strength are doubled compared with those of regular FaSSIF. In this way, after the addition of 25 mL of FaSSIF Concentrate to 25 mL of FaSSGF, the final dissolution medium will be the regular FaSSIF. The methodology and the preparation of the FaSSIF Concentrate are reported by J. Mann et al. [46]. Next, 2 mL aliquots were taken 1, 3, 5, 10, 15, 30, and 60 min after the medium change. Each taken volume was replaced with fresh FaSSIF media. The dissolution vessels were thermostated at 37 °C, and the paddle speed was set to 100 rpm during the experiment. Similar to the first release study, the cumulative drug release (~25 mg = 100%) was calculated from the absorbance values measured by UV-Vis spectrophotometry at a λ_max_ of 277 nm and 272 nm in the case of the FaSSGF and FaSSIF media, respectively. Each experiment was performed in triplicate, and the average values and standard deviations are reported.

#### 2.2.9. Study of Drug-Release Kinetics and Mechanism

The release kinetics of CIP from the electrospun nanofiber and physical mixture was compared with the dissolution kinetics of the CIP powder. Five different mathematical models (zero order, first order, Hixson–Crowell, Higuchi, and Korsmeyer–Peppas model) were fitted with the obtained cumulative drug release vs. time curves to describe the kinetics. To evaluate which model was followed, the value of the regression coefficient (*R*^2^) was determined and compared.

#### 2.2.10. In Vitro Diffusion Study

For the estimation of the passive diffusion of CIP through biological membranes, the Side-Bi-Side^TM^ (Crown Glass, Somerville, NJ, USA) diffusion test was carried out. The cellulose ester membrane (pore diameter = 0.45 µm) was dipped in isopropyl myristate before use. The donor phase was pH 6.8 PBS, and the acceptor phase was pH 7.4 PBS; both were kept at 37 °C temperature. The diffused drug content was measured in real-time at 272 nm by AvaLight DH-S-BAL spectrophotometer (AVANTES, Apeldoorn, The Netherlands) connected to an AvaSpec-2048L transmission immersion probe (AVANTES, Apeldoorn, The Netherlands). The optical path length was 1 cm. The diffusion tests of each sample were performed in triplicates, and the average values with the standard deviations are reported.

The flux, *J*, was calculated from the linear part of the graph using the following Equation (3):

*J* = ∂*m*/(*A* ∂*t*), (3)

where *m* is the cumulative amount of API transported in *t* time, and *A* is the surface area of the membrane (0.875 cm^2^). The permeability coefficient (*K_p_*) was determined by normalizing the flux to the donor concentration (*C_d_*), according to the Equation (4):

*K_p_* = *J*/*C_d_*, (4)

#### 2.2.11. Statistical Analysis

The significance levels of the differences between the measured fiber diameters of the samples produced under different parameters were examined by one-way analysis of variance (ANOVA) with post hoc Tukey HSD test. All the experimental results of solubility and single medium dissolution tests are expressed as mean ± standard deviation and statistically compared by a two-sample *t*-test. The experimental results with *p* values <0.05, <0.01, and <0.001 were assumed to be statistically significant.

## 3. Results and Discussion

### 3.1. Optimization of the Electrospinning Parameters

Reviewing the works focusing on the electrospinning of drug-loaded PVP nanofibers, the solution flow rate was found to range between 0.2 and 2 mL/h [23,36,37,38,39,40,41,42,43,44,45]. The average fiber diameter of the different formulations was largely varying. One of the aims of this research was to investigate the effect of flow rate on the diameter of PVP nanofibers, keeping the other preparation parameters constant. Additionally, nanofibers with different PVP:CIP volume ratios were prepared and studied. SEM was used to visualize the nanofibers, and then their morphology was observed, and the average fiber diameter was measured. The narrowest nanofiber formulation with the finest morphology was targeted during the optimization.

**Figure 2:**
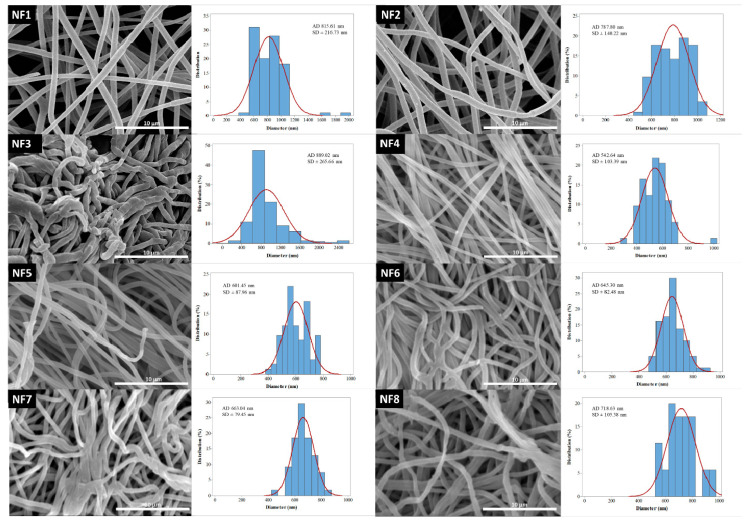
SEM images and diameter distributions of nanofibrous samples (5000× magnification).

The morphology of drug-free and various drug-loaded nanofibers are represented in Table 3 and Figure 2. Continuous, smooth-surfaced nanofibers were successfully prepared from all the solutions studied except for sample NF3. PVP:CIP 1:2 volume ratio eventuated discontinuous, worm-like nanofibers with largely varying diameters (889 ± 265 nm). The average diameter of the pure PVP fibers was 815 ± 216 nm. Considering the continuous fibers, the addition of the CIP decreased the fiber diameter. The decrease was significant except for sample NF2 (Table 4). This is expected to be related to the increase in the conductivity of the electrospinning solutions by adding the CIP [47]. Higher solution conductivity could facilitate the elongation of the jet and generate thinner fibers [48]. Additionally, the PVP nanofibers had some narrowing that slightly resembled the formation of beads, as shown in Figure 2. This was probably due to the low PVP concentration [49]. As the flow rate was increased, the average fiber diameter increased, which was expected from the literature [49]. However, the difference was not significant in every case (Table 4). Besides, the 3 mL/h flow rate (NF7) caused incomplete solvent evaporation and merged fibers, while the sample produced by 4 mL/h flow rate (NF8) showed some bead-like structures on top of the merged fibers (Figure 2). According to the results of this study, two formulations—namely, NF5 and NF6—had the most optimal morphology and diameter distribution. Thus, the 1:1 ratio between the PVP and CIP was found optimal, similar to our previous study [50]. The higher flow rate is desirable for the preparation of samples because of the larger number of nanofibers produced during the same period of time. Since the applied flow rate of NF6 was 2 times higher than the flow rate of NF5, finally, NF6 was selected for further studies.

### 3.2. Structural Characterization

**Figure 3:**
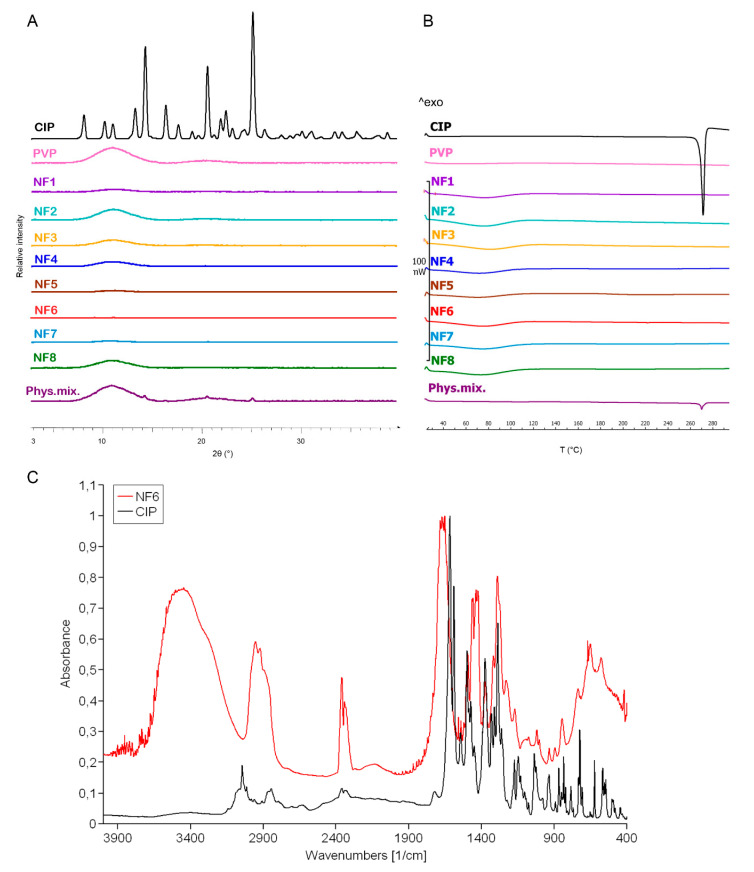
XRPD diffractogram (**A**) and DSC thermograms (**B**) of ciprofloxacin (CIP), PVP, physical mixture, and NF1–NF8 nanofibrous samples. All the electrospun samples are amorphous solid dispersions. FTIR spectra (**C**) of CIP and NF6 nanofibrous sample. The observed shifts and widenings confirm the successful incorporation of the CIP into the polymeric fibers.

The change in the crystallinity of CIP was studied by XRPD and DSC (Figure 3A,B). The XRPD of the CIP powder showed high crystallinity, represented by several smaller and three longer sharp peaks at around 2-Theta = 14.5, 20.9, and 25.4°. The diffractogram of the physical mixture also had the same peaks along with a broad peak between 2-Theta = 7–15°. The broad peak that appeared in the diffractogram of PVP, physical mixture, and the half of nanofibrous samples prove the polymer’s amorphous nature. The three characteristic peaks of the CIP were missing from the spectra of the nanofibrous samples, which indicates physicochemical interactions between the drug and the polymer matrix and the amorphous form of CIP created by the fast evaporation of the solvent during electrospinning.

Figure 3B presents the DSC thermograms of CIP, PVP, physical mixture, and all the samples listed in Table 2. The melting point of the pure drug is well-defined by a large endothermic peak at 275 °C, also appearing on the thermogram of the physical mixture. However, the flat PVP and NF1-NF8 thermograms suggest amorphous components. It can be concluded from both results that the drug was incorporated in amorphous form into the nanofibers.

Main drug–polymer interactions were observed by FTIR, and the spectra are given in Figure 3C. One prominent characteristic CIP peak at 1732 cm^−1^ (νC=O) shifted toward the higher wavenumbers and stretched due to the interaction with the hydroxyl groups of the PVP [51]. The wide band between 3050–3750 cm^−1^ (νC-OH) was enlarged by the hydrogen bonding between the CIP and PVP in the nanofibers. Moreover, the bands between 3050–2750 cm^−1^ (alkene and aromatic νC-H), between 2380–2275 cm^−1^ (νC-N), at 1500 cm^−1^ (quinolone), and at 1290 cm^−1^ (νC-O) appeared wider in case of the nanofiber [52]. These shifts and widenings confirm the successful incorporation of the CIP into the polymeric fibers.

### 3.3. Drug Loading and Drug Entrapment Efficiency

Nanofibers, in general, exhibit very high entrapment efficiency because the electrospinning procedure can be considered as an in situ solidification of a polymer solution. Since both CIP and PVP are non-volatile in nature, high entrapment efficiency was expected under the consideration of complete miscibility.

Entrapment efficiency, as well as drug loading, was calculated by UV spectrophotometry. Solvent with low pH (0.1 M HCl) was used to earn complete dissolution. The NF6 nanofibrous sample met our expectations with 92 ± 8% entrapment efficiency. Furthermore, the theoretical drug loading of the NF6 formulation was 0.99%, while the calculated drug loading was 0.92 ± 0.08%. These data are consistent with the high entrapment efficiency.

### 3.4. Solubility Test

The optimization study revealed that the sample NF6 had the most promising size and morphology for the solubility and dissolution studies. Solubility tests of the raw CIP powder, the physical mixture, and the drug-loaded nanofibrous samples were carried out in distilled water and PBS, as shown in Table 5. CIP has a pH-dependent, U-shaped solubility profile showing high solubility at pH < 5 and pH > 10 and poor solubility around neutral pH level as its isoelectric point is 7.42 [5]. Thus, distilled water and pH 7.4 PBS were chosen as solvents to observe the effect of the polymer nanocarrier on the solubility. The pH was measured at the beginning and the end of the tests. The pH values of the solvents before adding the CIP were 6.3 and 7.4 in the case of the distilled water and PBS, respectively. During the 24 h solubility study, the pH of the CIP solution in water increased to 7.1 because CIP as a weak base slightly alkalized the solution. With the increase of the pH level, the solubility of the drug decreases, which reduces the dissolution of the remaining solid CIP in the system. The buffer capacity of the PBS was sufficient to prevent a notable pH shift.

Studies with both solvents suggested that the incorporation of CIP into nanofibers caused a significant increase (*p* < 0.01 in the case of water and *p* < 0.05 in the case of PBS) in the drug solubility. In distilled water, the final CIP concentration showed a 12-fold increase in the case of nanofibers compared with the raw CIP, while in the PBS, the nanofibers were approx. 6.4 times more soluble. Furthermore, the solubility of the drug in the physical mixture was not significantly higher in either solvent. Thus, it can be seen that only the presence of PVP could enhance solubility by increasing wettability. However, this enhancement was much lower than in the case of nanofibers, where solid molecular dispersion of CIP formed during the electrospinning procedure. Solid molecular dispersions can guarantee increased solubility by decreasing the particle size and improving wettability. This finding may be correlated with the characterization results because inside the nanofiber the CIP is in its amorphous form.

### 3.5. In Vitro Drug Release

The in vitro release profiles of electrospun nanofibers and raw CIP powder were investigated both in single medium dissolution and two-stage biorelevant release tests. On the one hand, pH 7.4 PBS was chosen as a medium, while in this pH level, CIP has a minimum solubility value, and the pH of the terminal ileum [53], as well as the blood, is around 7.4. The pH-dependent solubility of a drug can cause incomplete dissolution or precipitation, which leads to suboptimal bioavailability. This might generate a problem with CIP because it occurs mostly at high administered doses [4]. On the other hand, any pH shift that occurs during the transfer from the stomach to the small intestine may affect the solubility and the bioavailability of CIP [54]. To investigate this effect, conditions similar to in vivo (biosimilar media, change in pH after 30 min, body temperature) were used by a two-stage release study [46].

**Figure 4:**
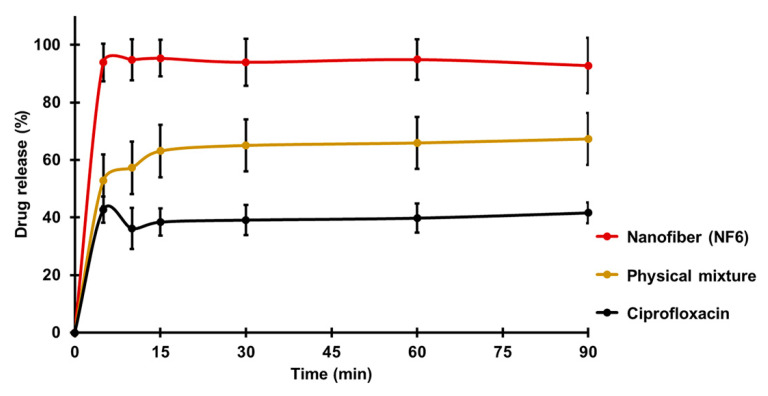
In vitro dissolution of ciprofloxacin (CIP) from NF6 nanofiber, physical mixture, and CIP powder in pH 7.4 phosphate buffer solution. All measured NF6 values were significantly higher than the raw CIP values (*p* < 0.001), and the first two were significantly higher than the physical mixture values (*p* < 0.05). Statistical analysis: Two-sample *t*-test.

Figure 4 presents the cumulative drug release vs. time curves of CIP powder, physical mixture, and electrospun sample (NF6) measured in PBS (pH 7.4). In the case of the CIP powder, the dissolution was not complete within 90 min, most probably due to the poor solubility of the drug at this pH. Until 90 min, only 41 ± 3% of the drug was liberated. Similarly, the drug release from the physical mixture was 67 ± 12% at the end of the measurement (90 min). However, NF6 showed a significantly higher dissolution rate than CIP powder (*p* < 0.001) at every measured point. Additionally, the nanofibrous sample demonstrated significantly higher drug release than the physical mixture (*p* < 0.05) in time points at 5 and 10 min. Besides, while all the samples showed fast dissolution behavior, the release was the fastest from the nanofibers. The improved dissolution rate resulted in 94 ± 6% dissolved CIP within only 5 min. The distinct difference between the raw CIP and the nanofibers could be caused by the high surface-to-volume ratio, the high wettability, and the amorphous drug inside the nanofibers.

**Figure 5:**
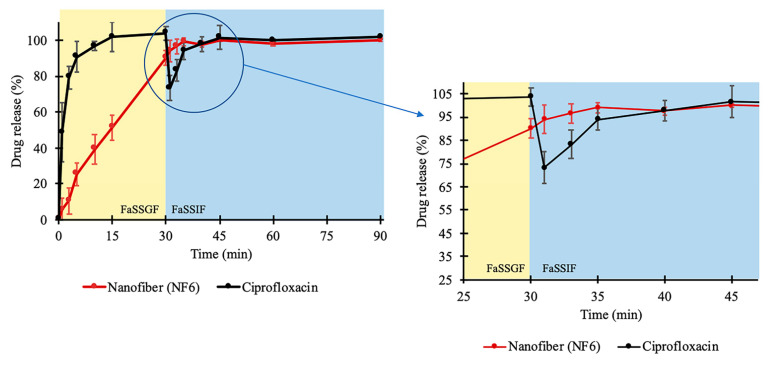
In vitro dissolution of ciprofloxacin (CIP) from NF6 nanofiber and CIP powder during the two-stage biorelevant release study. The dissolution medium (FaSSGF) simulated gastric condition until 30 min when the medium was turned into FaSSIF biorelevant fluid. The time range around the change of the medium (25–45 min) is magnified for better visualization.

In addition, a two-stage biorelevant release study was executed to mimic the in vivo conditions since the solubility of CIP is sensitive to changes in pH of the gastrointestinal environment. The raw CIP powder and the NF6 nanofibrous sample were placed into 25 mL of FaSSGF medium, and the drug release was studied for 30 min. From the raw CIP, the release was fast and complete as the whole amount of the added powder was dissolved within 10 min (Figure 5). This was expected since Hansmann et al. published a similar result with Ciprobay^®^ 500 mg IR tablet [54], and the 24 h solubility test in FaSSGF (described in Section 2.2.7) showed 7.794 ± 0.675 mg/mL average CIP concentration. However, in the case of the electrospun sample, the drug release was slower. It barely reached 90% until the last sampling of the FaSSGF medium. The difference could have been caused by the strong gelation of the high molecular weight PVP (Mw = 1,300,000) in low pH levels forming a viscous matrix around the CIP, which could remarkably slow down its diffusion to the dissolution medium.

As the second phase of the two-stage study, at 30 min, FaSSIF Concentrate was added to the FaSSGF dissolution medium to create a 1:1 volume ratio mixture (FaSSIF medium). With this step, the emptying of the stomach into the small intestine is modeled easily [54,55]. Due to the change of pH, the solubility of CIP decreased. As a result of the 24 h solubility test, 0.205 ± 0.002 mg/mL drug concentration was detected in FaSSIF (described in Section 2.2.7). In the case of the raw CIP samples, this 38-fold decrease caused the precipitation of the pre-dissolved CIP, which was visible in the vessels. Additionally, a slope can be observed at the 30 min time point in Figure 5. Interestingly, the curve of the nanofibrous sample has no slope, while the dissolution of the drug continued even after the change of the medium. Controlled release without precipitation was observed in the case of the electrospun sample. From the time point at 40 min, the two curves run together at around 100%. The time range between 25 and 45 min is magnified in Figure 5.

### 3.6. Drug-Release Kinetics and Mechanism

To describe the release kinetics from nanofibers, five different mathematical models are usually used—namely, zero order, first order, Hixson–Crowell model, Higuchi model, and Korsmeyer–Peppas model. The regression coefficient (*R*^2^) values of the different drug release models are listed in Table 6.

In the case of the single medium (pH 7.4 PBS) dissolution study, the Korsmeyer–Peppas model showed superiority over the other models studied for describing the release kinetic of the raw CIP, the physical mixture, and the NF6 electrospun sample. However, the Korsmeyer–Peppas model could not be fitted very well to the curves of CIP powder (*R*^2^ = 0.8967) and physical mixture (*R*^2^ = 0.8684), while the drug release of nanofibers was almost perfectly described by the model (*R*^2^ = 0.9993). This is reasonable since the Korsmeyer–Peppas model describes drug release from a polymeric system. It takes into account several mechanisms simultaneously, such as the diffusion of water into the polymer matrix, the swelling, and the dissolution of the polymer [52,56].

Furthermore, the Korsmeyer–Peppas model showed a high *R*^2^ value both in FaSSGF (*R*^2^ = 0.9794) and FaSSIF (*R*^2^ = 0.9229), considering the release kinetics of the two-stage biorelevant dissolution study. However, in the case of the latter, the first order kinetics could be fitted to the curve even more precisely (*R*^2^ = 0.9268). First order was found to be the release kinetics of the raw CIP in FaSSIF medium as well, which means that the dissolution rate of CIP from the PVP matrix was dependent on the drug concentration. This can be explained by the change of pH caused by the change of the medium at 30 min. The solution was supersaturated, and the higher pH caused the decrease of the solubility of the drug. Near to its solubility limit, the concentration of the CIP could affect the release kinetics.

In terms of the other medium, the release of the CIP powder followed the Higuchi model (*R*^2^ = 0.9502) while the highest *R*^2^ values of the nanofibrous sample were related to zero order (*R*^2^ = 0.9810) and the Hixson–Crowell model (*R*^2^ = 0.9873). The difference in the course of the curves can also be seen in Figure 5. The Higuchi model describes drug release from different matrix systems that contain water-soluble drugs [52]. The FaSSGF is a good solvent of the CIP and without any polymer in the system, it could dissolve freely. On the other hand, the PVP formed a viscous hemisphere gel at the bottom of the vessel, causing the Hixson–Crowell model. This release model considers the dissolution of a tablet or polymer matrix but with the maintenance of their geometrical characteristics.

### 3.7. In Vitro Diffusion Study

**Figure 6:**
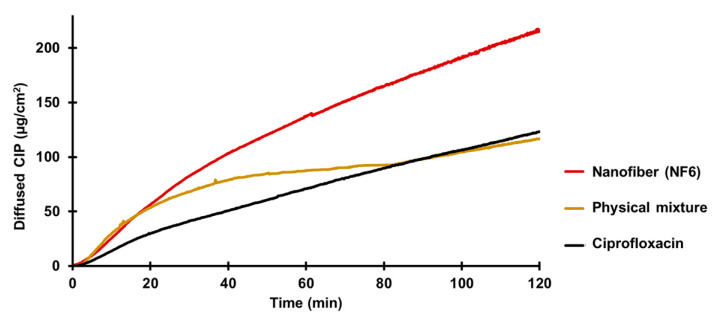
In vitro diffusion of ciprofloxacin (CIP) from the NF6 nanofibrous sample, physical mixture, and CIP powder (SD ± 2%).

An in vitro diffusion study was executed to compare the capacity of CIP from different samples (NF6 nanofiber, physical mixture, CIP powder) for crossing biological barriers, e.g., the small intestine cells (Figure 6). In the literature, it is suggested that the CIP is absorbed from the duodenum and the proximal jejunum [57]. Thus, during the in vitro diffusion study, pH 6.8 PBS was used for the donor phase. The acceptor phase (pH 7.4 PBS) modeled the intracellular pH (“set-point” pHi 7.35) of intestinal absorptive cells [58]. Since the absorption of ciprofloxacin seems to be mainly mediated not by active but by passive diffusion [54], a synthetic membrane was used to separate the two phases.

According to the results, the diffusion of the CIP was remarkably higher from the nanofibrous sample (215 µg/cm^2^) than the raw CIP (122 µg/cm^2^) or the physical mixture (118 µg/cm^2^). Figure 6 shows the diffusion profiles of CIP from the samples. The diffused CIP over time curve of the physical mixture interestingly runs with the curve of the nanofibrous sample for the first 20 min, but after that it becomes flatter as the CIP diffusion slows down. Finally, at the time range between 80 and 120 min, it overlaps with the curve of the pure CIP powder. This fascinating behavior could be caused by the presence of PVP. As written previously, PVP is a wetting agent that could increase the solubility of a drug. More solute CIP means a higher concentration gradient, which results in faster diffusion. However, the effect of this increase has a limit, and beyond that, the solution and diffusion of CIP slow down.

The calculated flux (*J*) and permeability coefficient (*K_p_*) values are shown in Table 7. The diffused CIP amount was 1.7 times higher from the NF6 sample than from the nonfibrous samples. The presence of the PVP did not affect the diffusion since the results of the physical mixture were similar to the CIP powders. Moreover, the permeability coefficient was increased 1.9 times by the nanofiber formulation.

## 4. Conclusions

Since CIP is a BSC Class IV drug, various pharmaceutical technological approaches are desirable for improving its bioavailability through the improvement in solubility, dissolution, and permeability. One such approach is the formulation of nanofibers via electrospinning, which is a simple and cost-effective production technique. Electrospun nanofibers, besides solid nanoparticles, are considered as amorphous solid dispersions and promising nanocarriers. Nanofibers are favorable drug carriers because of their high specific surface area, the wide variety of polymers and APIs that are spinnable, the ease of material combination, and the capability for mass production.

In the present study, CIP-loaded nanofibers were successfully fabricated by electrospinning. With the optimized process parameters, the nanofibers had a small, uniform fiber diameter with smooth surface morphology. The flow rates used in PVP-based nanofiber fabrication were compared. It was found that a higher flow rate produced thicker fibers, but in the case of too high a flow rate, the fibers were merged because the solvent evaporation was incomplete. According to the results of the XRPD and the DSC measurements, the CIP lost its crystallinity during the electrospinning procedure, and an amorphous form was produced. This form of the drug, along with the increased surface area, is the reason for the significantly higher solubility and in vitro dissolution rate in pH 7.4 PBS achieved with the nanofibrous samples. In the single medium release study (pH 7.4 PBS), the nanofibrous formulation demonstrated fast dissolution, and the release kinetics followed the Korsmeyer–Peppas model. In contrast, the raw CIP showed incomplete dissolution due to its poor solubility at this pH level. To mimic more precisely the in vivo conditions, a two-stage biorelevant release study was executed. Since the CIP is more soluble at low pH levels, a supersaturated solution was formed with FaSSGF medium. Then, with the change of the medium to FaSSIF, the solubility of the drug changed, and precipitation occurred. The precipitation could be prevented by nanofibers, since the PVP formed a viscous matrix around the CIP and released it with the dissolution kinetics described the best by Hixson–Crowell model and zero order kinetics. This study predicts a controlled release in the stomach for other API-loaded PVP-based nanofibers that might be useful in the development of local drug delivery systems. Moreover, incorporation of CIP into nanofibers could provide noticeably higher in vitro diffusion through the membrane. Therefore, our results show that CIP-loaded PVP-nanofibers could be considered as fast-dissolving formulations with improved physicochemical properties that may be suitable for further studies to keep in mind when developing an oral dosage form.

## Figures and Tables

**Table 1:** Ciprofloxacin-loaded nanocarriers under investigation with the aimed indication.

Type of Nanocarrier	Excipients Applied in Nanocarrier	Aimed Indications	Targeted Organs	References
Polymeric nanoparticles (NP) in nanofibers (NF)	PLGA and PCL—NP PEOT/PBT—NF	tissue engineering	middle ear	[15]
Nanoparticles and coated nanoparticles	PLGA and chitosan (coat)	root canal infection	tooth	[16]
Composite nanoparticles	synthetic nano-HA and sodium alginate	tissue engineering	bone	[17]
Microspheres	PLGA	osteomyelitis, orthopedic infections	bone	[18]
Microparticles	calcium carbonate, sodium hyaluronate	lung infections	lungs	[19]
Nanocrystals inside liposomes	HSPC, cholesterol	lung infections	lungs	[20]
Amorphous nanoparticle complex	dextran sulfate	non-cystic fibrosis bronchiectasis	lungs	[21]
Lipid-core nanocapsules	PCL, sorbitan monostearate, oleic acid, polysorbate 80	cystic fibrosis	lungs	[22]
Nanofibers	PVP	wound infections	skin	[23]
Nanofibers	PVA, chitosan, graphene oxide	wound infections	skin	[24]

Abbreviations: HA—hydroxyapatite; HSPC—hydrogenated soy phosphatidylcholine; PCL—poly(ε-caprolactone); PEOT/PBT—Poly(ethylene oxide terephthalate)/poly(butylene terephthalate) copolymer; PLGA—poly(d,l-lactide-*co*-glycolide); PVA—poly(vinyl alcohol); PVP—poly(vinyl pyrrolidone).

**Table 2:** The composition of each sample and their preparation procedures.

Sample	PVP Solution (*w*/*v* %)	PVP:CIP (*v*/*v*)	Flow Rate (mL/h)
NF1	5	1:0	2
NF2	20	1:3	2
NF3	15	1:2	2
NF4	10	1:1	0.5
NF5	10	1:1	1
NF6	10	1:1	2
NF7	10	1:1	3
NF8	10	1:1	4

**Table 3:** Fiber morphology and average diameter for different formulations.

Sample	Fiber Morphology		Average Diameter (nm)
NF1	continuous	smooth	815 ± 216
NF2	continuous	smooth	787 ± 140
NF3	discontinuous	worm-like	889 ± 265
NF4	continuous	smooth	542 ± 103
NF5	continuous	smooth	601 ± 87
NF6	continuous	smooth	645 ± 82
NF7	continuous	smooth	663 ± 79
NF8	continuous	rough	718 ± 105

**Table 4:** Q statistic values of the different nanofibrous formulations as pairs of treatments in one-way ANOVA with post hoc Tukey HSD test. The significantly different (** *p* < 0.01; * *p* < 0.05) pairs of treatments are marked.

Sample	NF1	NF2	NF3	NF4	NF5	NF6	NF7	NF8
NF1	-	1.77	4.54 *	15.47 **	12.54 **	9.91 **	7.88 **	4.31 *
NF2	1.77	-	6.45 **	14.24 **	11.21 **	8.51 **	6.58 **	3.12
NF3	4.54 *	6.45 **	-	19.67 **	16.88 **	14.21 **	11.69 **	7.58 **
NF4	15.47 **	14.24 **	19.67 **	-	3.19	5.53 **	5.85 **	7.46 **
NF5	12.54 **	11.21 **	16.88 **	3.19	-	2.43	3.07	5.05 **
NF6	9.91 **	8.51 **	14.21 **	5.53 **	2.43	-	0.88	3.15
NF7	7.88 **	6.58 **	11.69 **	5.85 **	3.07	0.88	-	2.23
NF8	4.31 *	3.12	7.58 **	7.46 **	5.05 **	3.15	2.23	-

**Table 5:**
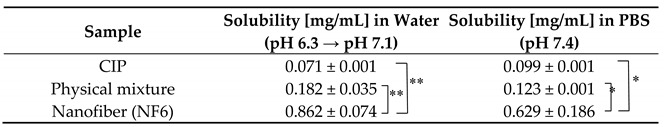
Solubility data of the samples in distilled water and phosphate buffer. The dissolution of the ciprofloxacin (CIP) shifted the pH from 6.3 to 7.1 in distilled water. The nanofiber NF6 has significantly higher (** *p* < 0.01; * *p* < 0.05) solubility than the physical mixture and the raw CIP powder. Statistical analysis: Two-sample *t*-test.

**Table 6:** Regression coefficient values of the different drug release models.

Release Study	Single Medium (pH 7.4 PBS)			Two-Stage Biorelevant (FaSSGF)		Two-Stage Biorelevant (FaSSIF)	
Sample	CIP	Physical Mixture	Nanofiber (NF6)	CIP	Nanofiber (NF6)	CIP	Nanofiber (NF6)
Zero order	0.3270	0.2955	0.6122	0.5820	0.9810	0.2687	0.2344
Fist order	0.8463	0.6565	0.9807	0.9085	0.9602	0.9276	0.9268
Hixson–Crowell	0.3449	0.3540	0.6431	0.9204	0.9873	0.6452	0.5282
Higuchi	0.8908	0.8632	0.9577	0.9502	0.9616	0.8273	0.8304
Korsmeyer–Peppas	0.8967	0.8684	0.9993	0.8895	0.9794	0.8717	0.9229

**Table 7:** Calculated flux (*J*) and permeability coefficient (*K_p_*) values.

Sample	*J* (µg/(cm^2^ h))	*K_p_* (cm/h)
CIP	60.94	0.112
Physical mixture	58.80	0.105
Nanofiber (NF6)	107.71	0.217

## Data Availability

The datasets used and/or analyzed are available from the corresponding author on reasonable request.
